# Kynurenic acid in neurodegenerative disorders—unique neuroprotection or double‐edged sword?

**DOI:** 10.1111/cns.13768

**Published:** 2021-12-03

**Authors:** Aleksandra Ostapiuk, Ewa M. Urbanska

**Affiliations:** ^1^ Laboratory of Cellular and Molecular Pharmacology Department of Experimental and Clinical Pharmacology Medical University of Lublin Lublin Poland; ^2^ Present address: Department of Clinical Digestive Oncology KU Leuven Leuven Belgium

**Keywords:** Alzheimer's disease, Huntington's disease, mitochondrial toxin, neurodegeneration, N‐methyl‐D‐aspartate, Parkinson's disease

## Abstract

**Aims:**

The family of kynurenine pathway (KP) metabolites includes compounds produced along two arms of the path and acting in clearly opposite ways. The equilibrium between neurotoxic kynurenines, such as 3‐hydroxykynurenine (3‐HK) or quinolinic acid (QUIN), and neuroprotective kynurenic acid (KYNA) profoundly impacts the function and survival of neurons. This comprehensive review summarizes accumulated evidence on the role of KYNA in Alzheimer's, Parkinson's and Huntington's diseases, and discusses future directions of potential pharmacological manipulations aimed to modulate brain KYNA.

**Discussion:**

The synthesis of specific KP metabolites is tightly regulated and may considerably vary under physiological and pathological conditions. Experimental data consistently imply that shift of the KP to neurotoxic branch producing 3‐HK and QUIN formation, with a relative or absolute deficiency of KYNA, is an important factor contributing to neurodegeneration. Targeting specific brain regions to maintain adequate KYNA levels seems vital; however, it requires the development of precise pharmacological tools, allowing to avoid the potential cognitive adverse effects.

**Conclusions:**

Boosting KYNA levels, through interference with the KP enzymes or through application of prodrugs/analogs with high bioavailability and potency, is a promising clinical approach. The use of KYNA, alone or in combination with other compounds precisely influencing specific populations of neurons, is awaiting to become a significant therapy for neurodegenerative disorders.

## Graphical abstract

Kynurenic acid (KYNA), metabolite of tryptophan along the kynurenine pathway, exhibits neuroprotective activity under *in vitro* and *in vivo* conditions. As novel KYNA targets were identified, distinctive biological role of the compound was recognized. Absolute or relative deficiency of KYNA in proportion to neurotoxic kynurenines in Huntington's, Parkinson's, and Alzheimer's diseases implicates potential therapeutic value of boosting brain KYNA levels.

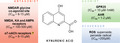

## 1 INTRODUCTION

**FIGURE 1:**
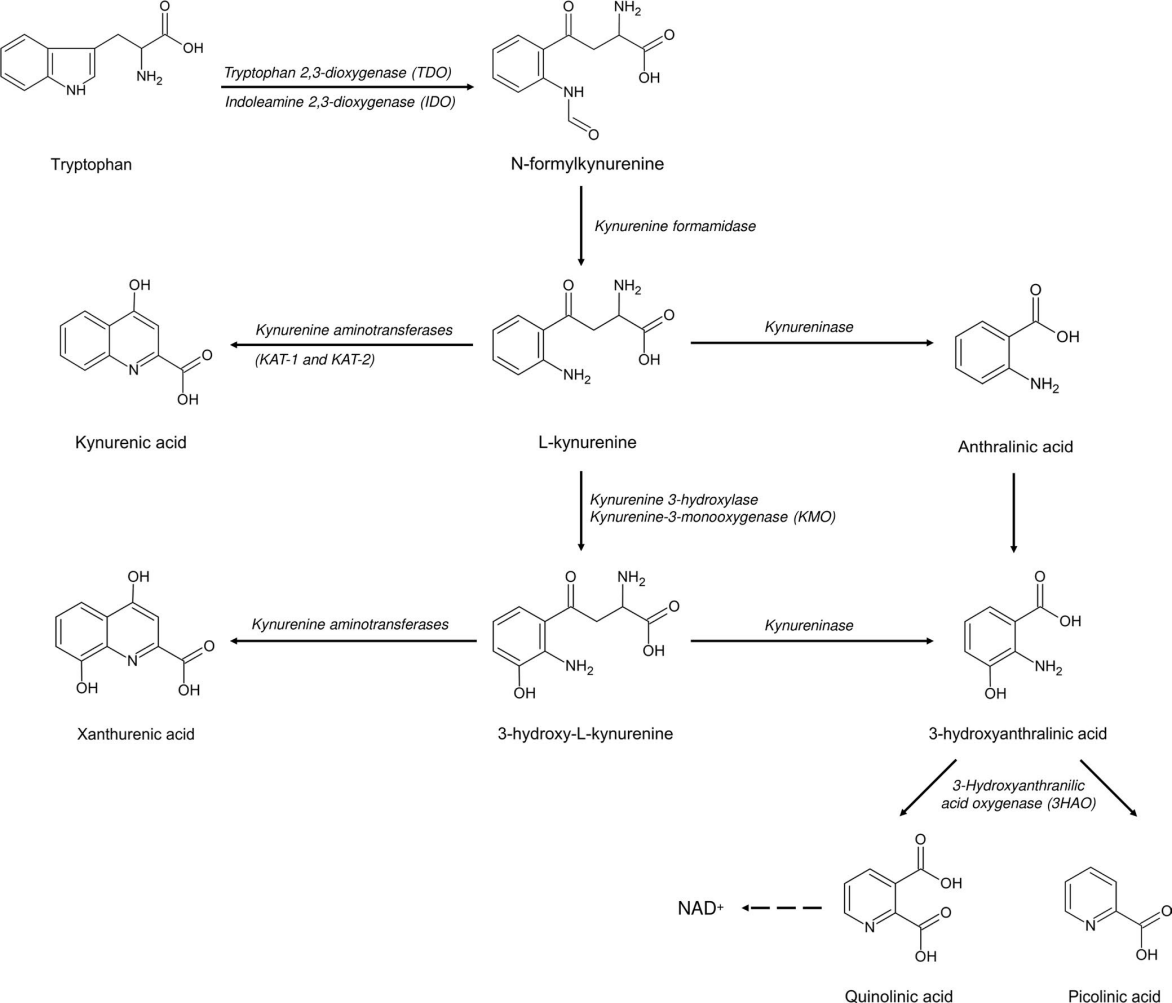
Scheme of kynurenine pathway

Tryptophan, an essential neutral amino acid, is a pivotal constituent of proteins and a source of numerous, biologically significant compounds. Only a small quantity of compound (1–2%) undergoes incorporation into peptides or proteins, whereas the remaining 98–99% follows two major metabolic routes. These include (a) the methoxyindole pathway yielding 5‐hydroxytryptamine (5‐HT; serotonin), and (b) the kynurenine pathway (KP) generating metabolites collectively called kynurenines (Figure 1). The KP converts 95% of tryptophan and ultimately leads to the formation of nicotinamide adenine dinucleotide (NAD+), with a number of neuroactive kynurenines en route.^1^, ^2^ The discoveries of last decades strongly support the concept of viewing the disturbed KP as an important link in the cycle of events leading to the development of brain pathologies. Various kynurenines are of substantial biological importance due to their ability to modify neurotransmission and to alter the immune response.^3^, ^4^

The family of KP metabolites comprises compounds acting in a divergent way and considered to be either neuroprotective or neurotoxic.^5^ The synthesis of specific compounds is tightly regulated and may considerably vary under physiological and pathological conditions.^5^ Kynurenic acid (KYNA), the main neuroprotective compound of the path, was discovered in the 19^th^ century as a constituent of canine urine and initially regarded merely as a by‐product of tryptophan degradation.^6^ The molecular structure of KYNA was unraveled at the beginning of the 20^th^ century,^7^ yet the particular steps of the KP leading to KYNA formation were determined much later. Discoveries of the 1980s revealed the ability of KYNA to block the excitatory amino acid receptors under *in vitro* and *in vivo* conditions.^8^, ^9^ Soon, abnormalities in cerebral KYNA synthesis have been implicated in the pathogenesis of neurodegeneration.^10^ Intensified research during last four decades revolutionized our knowledge about KYNA and brought valuable data supporting the significant role of KYNA as an exceptional tryptophan metabolite in the mammalian brain.^11^ This review aimed to discuss the involvement of altered KYNA metabolism in the development of neurodegenerative diseases, as well as the future of pharmacological manipulations aimed to boost brain KYNA as potential therapeutic agents.

The KP is functional in the brain and in the periphery.^12^ The first step of tryptophan metabolism is catalyzed by the step‐limiting enzymes, indoleamine 2,3‐dioxygenases (IDO‐1 and 2) and tryptophan 2,3‐dioxygenase (TDO), yielding N‐formyl‐kynurenine (Figure 1). N‐formyl‐kynurenine is further converted to a direct precursor of KYNA, L‐kynurenine, by formamidase. In the periphery, the constitutive expression of IDO‐1 is restricted and was described mostly within endothelial, pancreatic, placental, or antigen‐presenting cells. Interestingly, IDO‐1 manifests pronounced susceptibility to the induction by proinflammatory molecules, such as interferon‐γ, tumor necrosis factor α (TNF‐α), interleukin‐6 (IL‐6), or IL‐10, in a variety of cells.^13^, ^14^

IDO‐2 and TDO show higher tissue specificity, mainly restricted to liver, and much lower expression level.^15^ The enzymatic activity of TDO can be induced by estrogens, glucocorticoids, and tryptophan itself.^2^ In the brain, striatal neurons and astrocytes express high levels of IDO‐1 mRNA.^16^ TDO protein and its mRNA are also detectable in neurons and astrocytes.^17^, ^18^, ^19^

The major central pool of KYNA is formed locally, from its precursor, L‐kynurenine.^20^, ^21^ L‐kynurenine, on the contrary, originates mostly from peripheral sources (60–70%), whereas the remaining 30–40% is produced *in situ*.^21^ L‐kynurenine can be also converted along another arm of the KP to neurotoxic 3‐hydroxykynurenine (3‐HK), QUIN, and further down to NAD.^22^ The fate of L‐kynurenine degradation and its availability for the synthesis of KYNA is determined by a number of factors, including tissue and cell type. Central KYNA production occurs mostly in astrocytes and endothelial cells and to a much lesser extent within neurons.^23^, ^24^, ^25^, ^26^ In contrast, neurotoxic QUIN is generated in the human brain mainly by the microglial cells and macrophages.^27^

The principal route of KYNA synthesis is based on an irreversible transamination of L‐kynurenine catalyzed by kynurenine transaminases (KATs).^28^ KYNA is produced by various tissues and organs, including liver, kidneys, intestines, or endothelium.^29^, ^30^ In the brain, KATs are expressed mainly in astrocytes and to a lesser degree in neuronal cells, for example, in hippocampus, substantia nigra, or striatum.^24^, ^25^, ^31^, ^32^, ^33^ KATs are characterized by a different level of specific activity in various brain regions.^34^, ^35^

In humans and rodents, four isoforms of KATs, using L‐kynurenine as a donor for amino group, were characterized and include KAT I (glutamine transaminase K/cysteine conjugate beta‐lyase 1), KAT II (α‐aminoadipate aminotransferase), KAT III (glutamine transaminase L/cysteine conjugate beta‐lyase 2), and KAT IV (the mitochondrial aspartate aminotransferase/glutamic‐oxaloacetic transaminase 2).^36^ Each KAT enzyme has an optimal pH range and a distinct substrate profile, despite sharing a number of amino acid and α‐keto acid substrates.^37^, ^38^, ^39^ KATs manifest relatively low affinity for L‐kynurenine (K_m_ approx. 1 mM). Under physiological conditions, KAT II is considered a major biosynthetic enzyme responsible for KYNA formation.^40^

A targeted deletion of KAT II in mice leads to an early and transitory decrease in brain KAT activity and KYNA levels with commensurate behavioral and neuropathological changes.^41^ In KAT II–deficient mice, striatal KYNA level was transiently reduced around the 2^nd^ week of age and the degree of neuronal loss following the local administration of QUIN was strongly enhanced.^42^ Later on, however, KYNA levels were normalized, possibly as a result of compensatory changes.^41^, ^42^

Indirectly, the activity of kynurenine monooxygenase (KMO), synthesizing 3‐HK and displaying a much lower K_m_ value for L‐kynurenine, also impacts the synthesis of KYNA. Inhibition of KMO activity increases the pool of L‐kynurenine available for KATs. This, in turn, may easily shift the KP and direct it to the neuroprotective branch; conversely, an enhanced activity of KMO stimulates metabolism of tryptophan along the neurotoxic arm of the pathway.^43^

As shown under *in vitro* and *in vivo* conditions, the composition of the extracellular milieu, the availability of oxygen and glucose, or level of ammonia and amino acids may influence the synthesis of KYNA.^44^, ^45^, ^46^, ^47^ Notably, neurotoxic compounds such as mitochondrial toxins or pyrethroid pesticides inhibit, whereas a number of therapeutic agents, including beta‐adrenoceptor agonists, nitric oxide donors, memantine, antidepressants, or some antiepileptics, stimulate KYNA production in the brain ^48^, ^49^, ^50^, ^51^, ^52^, ^53^.

### 1.1 Other sources of brain KYNA

Apart from the canonical KAT‐related synthesis, alternative mechanisms were implicated in the synthesis of KYNA.^54^, ^55^ Indole‐3‐pyruvic acid, the keto‐analog of tryptophan, increases KYNA content in various organs, including brain.^56^ Indole‐3‐pyruvic acid is effectively converted to KYNA in a non‐enzymatic reaction requiring ample presence of oxygen. Reactive oxygen species (ROS) target the enol form of indole‐3‐pyruvic acid, which undergoes pyrrole ring cleavage and subsequently forms KYNA.^57^, ^58^ L‐L‐kynurenine may also yield KYNA when incubated in the presence of H_2_O_2_, with or without peroxidases.^55^ It is a pH‐dependent process, with the highest conversion of L‐kynurenine occurring at the pH between 7.4 and 8.0.^55^ The contribution of alternative routes to the overall KYNA production still remains unclear. However, in the altered redox environment and when the antioxidant system is defective, as often is the case in neurodegenerative disorders, their significance may increase. Indeed, the lack of correlation between KATs activities and KYNA levels was reported in lead intoxication, Down syndrome, and disturbances of thyroid hormone levels.^59^, ^60^, ^61^, ^62^

Furthermore, although peripherally synthesized KYNA poorly passes through the blood‐brain barrier, certain conditions may facilitate its penetration into the brain. Systemic administration of KYNA prior to the cerebral ischemia potently increased its brain concentrations,^63^ possibly as a result of passive diffusion.^21^ In addition, KYNA was identified as a high‐affinity substrate for organic anion transporters, OAT1 and OAT3.^64^, ^65^ Experimental use of probenecid, a non‐selective inhibitor of Oat1, was shown to increase the brain level of KYNA.^66^, ^67^ Interestingly, thyroid hormones may enhance the removal of KYNA and modulate its brain level via diverse mechanisms, including the action of Oat.^59^

Finally, apart from the *de novo* synthesis by the mammalian tissues, KYNA can be generated in the digestive system by microbiota and exogenously delivered with food products.^68^, ^69^

### 1.2 Neurotoxic branch of kynurenine pathway

Three major neurotoxic metabolites of the KP include 3‐HK, QUIN, and 3‐hydroxyanthranilic acid (3‐HANA). 3‐HK is an immediate product of L‐kynurenine conversion carried out by KMO. Metabolism of 3‐HK by kynureninase yields 3‐HANA, which, in two enzymatic steps, can be further converted to QUIN. The toxicity of 3‐HK has been attributed mainly to the formation of free radicals and an induction of apoptotic neuronal death.^70^, ^71^

The results of numerous research clearly indicate that QUIN is capable of acting as an endogenous excitotoxin. QUIN‐evoked neuronal loss is mostly associated with an excessive stimulation of NR2A and NR2B subunits of N‐methyl‐D‐aspartate (NMDA) receptor at agonist‐binding site. In the brain, physiological concentrations of QUIN are in nM range (~50–100 nM) and are approx. 20 times lower than in the periphery.^72^ At low concentrations, QUIN induces proliferation of the stem cells and is an intermediate metabolite along the pathway yielding NAD+in human brain cells.^73^, ^74^ At high, close to millimolar concentrations, QUIN induces selective, axon‐sparing neuronal loss under various experimental conditions. The susceptibility of neurons to the QUIN‐induced damage depends on the brain area, with cortical, striatal, and hippocampal neurons being the most sensitive.^3^ It was debated whether endogenous levels of QUIN are sufficient to cause neurotoxicity, yet, in the view of accumulated data, the compound undoubtedly may evoke neuronal death. In human brain, QUIN levels increase during inflammation or cerebral insults up to the micromolar values.^75^ Locally, QUIN concentration may be much higher.^3^ Notably, even low concentrations of QUIN may induce neuronal loss, providing that the exposure is prolonged. In organotypic corticostriatal, but not in caudate nucleus, cultures, exposed to low (100 nM) concentration of QUIN for up to 7 weeks, a clear focal neurodegeneration was developed.^76^

QUIN was also demonstrated to enhance the glutamate release, to inhibit glutamate reuptake, and to stimulate lipid peroxidation.^77^ Such local rise evokes depolarization of the postsynaptic membrane sufficient to remove the Mg^2+^ block of NMDA receptor–linked ion channel. Moreover, QUIN may impair the function of blood‐brain barrier, induce nitric oxide production, and cause hyperphosphorylation of cytoskeletal intermediate filament proteins in astrocytes and neurons.^73^, ^78^, ^79^, ^80^ In the view of accumulated data, QUIN produces neurotoxicity through a link of events initiated by the excessive stimulation of the NMDA receptor, Ca^2+^ influx, energy deficit, and oxidative stress.

The proper balance between neurotoxic kynurenines and neuroprotective KYNA can be viewed, in fact, as the interplay between astrocytes, microglia, and neurons. Under physiological conditions, the astrocytic KP serves as a source of neuroprotective KYNA, whereas neuronal KP produces NAD+ improving cellular energy status. In diseased brain, the inflammatory signals stimulate the KP within macrophages, microglia, and dendritic cells to produce large quantities of QUIN. Astrocytes remove QUIN from synaptic cleft and catabolize it to NAD+; however, the enzyme responsible for catabolism is easily saturated. Thus, when the overall balance of the pathway is shifted toward QUIN and other neurotoxic kynurenines, with an absolute/relative deficiency of KYNA, neurodegenerative changes can follow.

## 2 BIOLOGICAL KYNA TARGETS IN THE BRAIN

The level of KYNA in the central nervous system (CNS) depends on the species, studied region, and the ontogenetic stage of development.^81^ In human brain, KYNA occurs in low micromolar range (approx. 0.1–1.5 μM), which is 20–50 times higher than in rodent CNS (0.001–0.05 μM).^34^, ^82^, ^83^, ^84^, ^85^ The content of KYNA was reported to be the lowest in cerebellum and medulla (0.1–0.3 pmol/mg), intermediate in cortical areas and substantia nigra (0.2–0.6 pmol/mg), and the highest in putamen and globus pallidus (0.7–1.4 pmol/mg).^34^, ^82^ In human CSF, KYNA concentration is low (0.001–0.01 μM), yet it steadily increases with age.^81^, ^86^, ^87^ In other species, the brain content of KYNA also rises with age.^88^ Over 50‐fold increase in brain KYNA between 1^st^ week and 18^th^ month of age was reported in rats.^89^ Others demonstrated a threefold increase between the 3^rd^ and 24^th^ months of age.^90^

**FIGURE 2:**
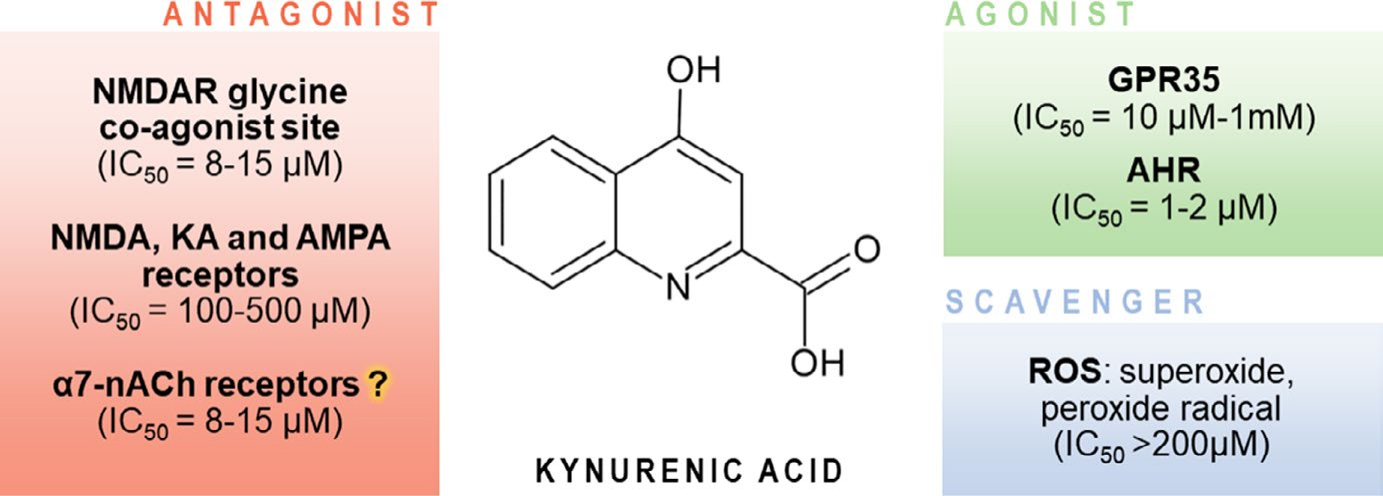
Targets of kynurenic acid. NMDA—N‐methyl‐D‐aspartate; KA—kainic acid; AMPA—alpha‐amino‐3‐hydroxy‐5‐methyl‐4‐isoxazolepropionic acid; AHR—aryl hydrocarbon receptor; GPR35—G protein–coupled orphan receptor 35; ROS—reactive oxygen species

KYNA is quickly liberated from the cell and is not a subject of enzymatic degradation or reuptake processes.^11^, ^91^ Extracellular KYNA interacts with a number of biological targets (Figure 2). KYNA was initially recognized as a broad‐spectrum antagonist of ionotropic excitatory amino acid receptors. KYNA displays the highest affinity for the co‐agonist glycine site of NMDA receptor complex (IC_50_ ~8–15 μM in the absence of glycine; ~50–200 μM in the presence of 10 μM glycine.^92^, ^93^ KYNA, in a competitive manner, blocks also agonist‐binding sites of NMDA, kainic acid, and alpha‐amino‐3‐hydroxy‐5‐methyl‐4‐isoxazolepropionic acid (AMPA) receptors, yet with lower affinity (IC_50_ of 100–500 μM).^3^, ^94^ Despite the discrepancy between physiologically occurring levels of KYNA and the levels needed to interfere with glutamatergic receptors, it is well established that KYNA synthesis may increase locally due to various factors and easily reach the concentration sufficient to interact with NMDA receptors.^95^

Neuroprotective and anticonvulsant properties of KYNA are broadly documented under *in vivo* and *in vitro* conditions.^94^, ^95^, ^96^, ^97^ Notably, KYNA attenuates the morphological and behavioral consequences of experimental administration of its kynureninergic *alter ego*—excitotoxic QUIN.

As KYNA may impact the extracellular levels of glutamate, acetylcholine, GABA, and dopamine, neuromodulation is an important aspect of its role.^98^, ^99^, ^100^, ^101^ In striatal preparations, low nanomolar concentrations of KYNA reduced glutamate release in caudate nucleus and impaired the neurotransmitter release.^102^ Experimental studies *in vivo* confirmed that fluctuations of KYNA level may alter glutamine, acetylcholine, and dopamine release.^98^, ^99^, ^101^

KYNA has also been identified as a ligand of formerly orphan G protein–coupled receptor, GPR35,^103^ broadly expressed in various immune cells. Apart from the regulation of immune response, KYNA‐GPR35 interaction may inhibit Ca^2+^ channels in sympathetic neurons and reduce synaptic activity in hippocampal neurons.^104^, ^105^ Therefore, KYNA capability to activate GPR35 might represent another way to reduce the excitatory transmission.^105^, ^106^ KYNA is also targeting xenobiotic receptor, the aryl hydrocarbon receptor (AHR).^107^ KYNA‐related AHR stimulation increases the interleukin‐6 expression, which is often associated with promoting carcinogenesis and tumor outgrowth.^107^, ^108^ Moreover, KYNA displays the scavenging ability toward ROS. In the homogenates of rat brains, KYNA decreased the production of free radicals and lipid peroxidation.^109^ It has been postulated that KYNA targets also α7‐nicotinic acetylcholine receptors (α7nAChR); however, this mechanism is still being controversial.^110^, ^111^, ^112^

**FIGURE 3:**
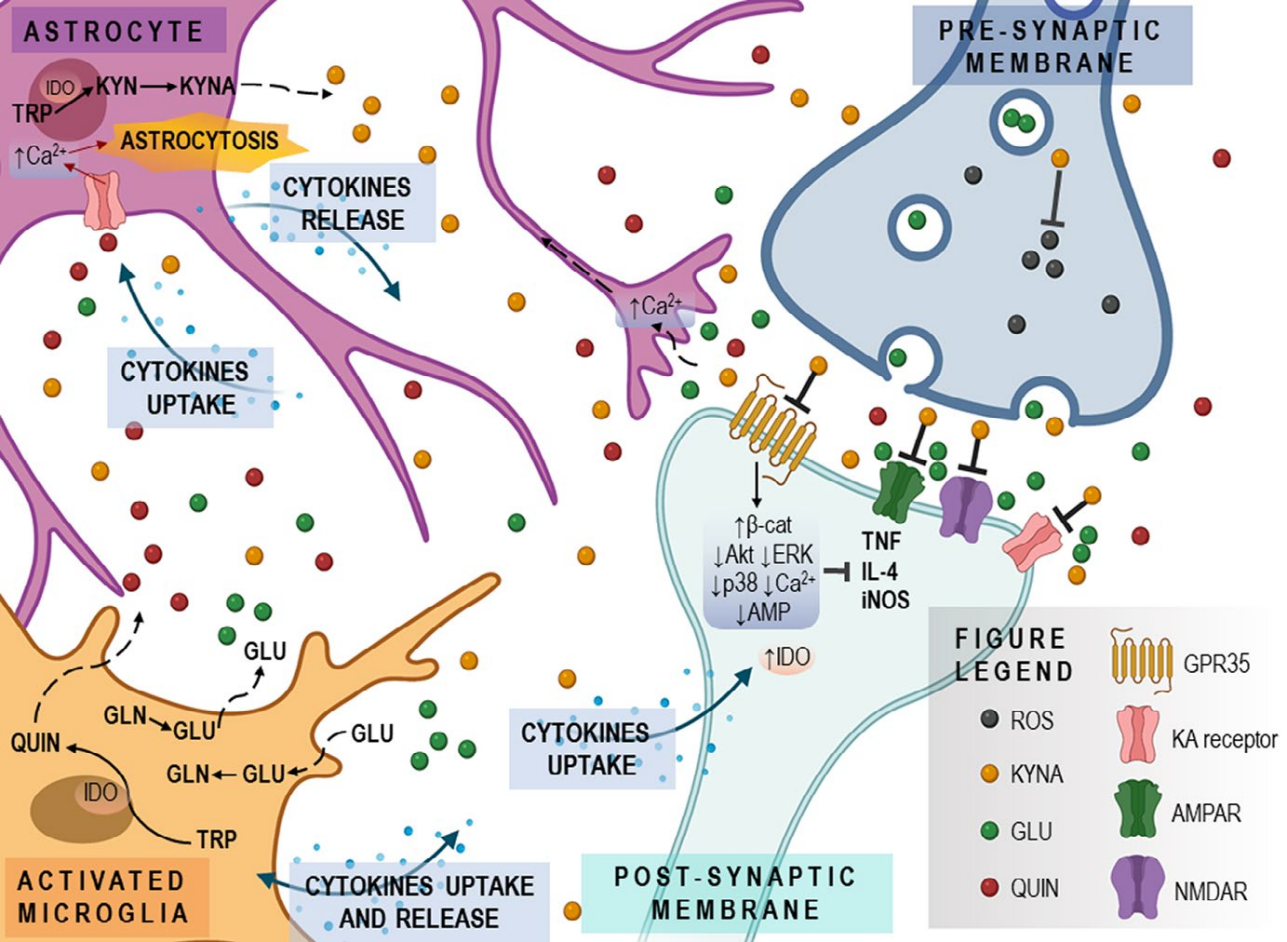
Role of kynurenic acid in neurodegeneration. The interplay between astrocytes, microglia, and neurons in terms of the quantities of produced KYNA and other kynurenines can be altered by various genetically determined and postnatal factors, including inflammation. Deficiency of KYNA may enhance the GLU‐mediated neurotransmission, reduce antioxidant capacity, and shift the kynurenine pathway toward neurotoxic metabolites, with ensuing neuronal loss

The inflammation emerged as one of the key factors contributing to the neuronal loss and compromised regeneration and thus was implicated in the pathogenesis of neurodegenerative disorders. An important link between proinflammatory status and the activation of KP is well substantiated.^11^, ^86^ Importantly, the metabolites of KP may act as pro‐ and antiinflammatory compounds. Genomic interventions aimed to eliminate IDO, TDO, or KMO were shown to alleviate the course of chronic inflammation, reduce viral replication, or change the expression of proinflammatory molecules.^113^ On the contrary, a number of kynurenines, including KYNA, emerged as antiinflammatory compounds.^11^, ^113^ KYNA was demonstrated to attenuate inflammation by several ways including the reduction in TNF expression, diminished interleukin‐4 and α‐defensin secretion, or inhibition of Th17 cell differentiation, at least in part through activation of GPR35.^11^ The interplay between immune activation and the KP activity results in a delicate balance, which may easily be shifted either to or away from neuroprotective KYNA (Figure 3).

However, an excessive blockade of glutamate‐mediated neurotransmission may impair cognition and memory processes.^114^, ^115^, ^116^, ^117^, ^118^ Thus, manipulations of the endogenous KYNA level may exert dual, conflicting effects—beneficial neuroprotection and unfavorable cognitive dysfunction. Considering the chronic nature of neurodegenerative disorders, neuroprotection seems to be essential, as it may slow the progress of disease. Maintaining adequate levels of brain KYNA seems vital to obtain neuroprotection without cognitive adverse effects. The optimal therapeutic intervention would include a region‐selective increase in KYNA; however, such pharmacological tools are not available yet.

## 3 KYNA ALTERATIONS IN NEURODEGENERATIVE DISEASES

### 3.1 Huntington's disease

Huntington's disease (HD) is an autosomal dominant neurodegenerative disease. Clinically, it is characterized by a gradual deterioration of voluntary movements, appearance of chorea, cognitive decline, and complex psychiatric symptoms.^119^ Symptoms begin slowly, usually in the fourth decade of life, and lead ultimately to death within 15–20 years. The genetic background of disease is linked with an expansion of CAG trinucleotide repeats within exon 1 on chromosome 4, following a single mutation within the *IT15* gene encoding huntingtin.^120^ Neurodegeneration affects primarily cerebral cortex and striatum, but as disease progresses, neuronal loss develops in multiple areas of the brain. Apart from the accumulation of huntingtin, the precise mechanisms leading to neurodegeneration and subsequent clinical symptoms are not fully elucidated. Aberrations in function of glial cells, inflammation, mitochondrial dysfunction, or oxidative stress were all implicated in the pathogenesis of HD.^121^, ^122^, ^123^

The potential role of aberrant tryptophan metabolism in the pathogenesis of neurodegenerative disorders has been postulated by Schwarcz and co‐workers who discovered that intrastriatal application of excitotoxic KP metabolite, QUIN, results in neuropathological and behavioral alterations closely mimicking HD.^124^ Further support to this concept was provided by numerous research data, including studies on the experimental KYNA deficiency caused by the pharmacological tools, aminooxyacetic acid (AOAA), and 3‐nitropropionic acid (3‐NPA).^96^, ^125^, ^126^ AOAA, a non‐selective aminotransferase inhibitor, potently diminishes synthesis of KYNA *in vitro*, with very low, micromolar IC_50_ values. When administered intrastriatally, AOAA produces a pattern of neurodegenerative and behavioral changes modeling HD and astonishingly resembling the outcome of intrastriatal application of QUIN.^96^ The axon‐sparing excitotoxic neuronal loss is age‐dependent, is susceptible to blockade with KYNA itself, and could be prevented by the ablation of corticostriatal glutamatergic input.^96^

Similarly, 3‐NPA was shown to impair the synthesis of KYNA in rat cortical slices and to inhibit the activity of KAT I and KAT II.^126^ *In vivo*, 3‐NPA decreased the number of KAT I immunopositive glial cells in the striatum (−3.57‐fold) and temporal cortex (‐twofold) of rats.^127^ Behavioral consequences of 3‐NPA application in rodents are influenced by the mode of treatment. Acute application of 3‐NPA evokes seizures, whereas chronic administration of low doses of 3‐NPA results in a progressive locomotor deterioration and selective striatal degeneration resembling changes characteristic for HD.^128^, ^129^, ^130^ Susceptibility to the effects of 3‐NPA increases with age; furthermore, 3‐NPA and mutated huntingtin seem to share certain mechanisms of toxicity.^131^

Alterations in the metabolism of KP have also been demonstrated in genetic animal models of HD. In FVB/N mice with a mutation in the huntingtin gene, more than 10‐fold increase of 3‐HK level in the striatum and cortex was accompanied by a slight increase in KYNA levels and a considerable, 5.7‐fold, increase in the 3‐HK/KYNA ratio.^132^ A study in R6/2 mice, modeling HD, also demonstrated the increased activity of KMO (1.65‐fold change in v_max_ value between 8‐week‐old wild‐type and R6/2 animals) and decreased activity of kynureninase (−1.5‐ to −1.67‐fold), resulting in an excessive enzymatic conversion of tryptophan to 3‐HK.^133^

Data from human studies are in line with the experimental research, despite a clear limitation of *postmortem* analyses. Brain changes in KYNA content seem to be region‐selective. Neostriatal KYNA level was reported as either decreased or unaltered, whereas cortical KYNA levels seem to increase, especially during the late stage of disease.^34^, ^83^, ^134^, ^135^, ^136^ Furthermore, an increase in frontocortical QUIN and 3‐HK (both c. 2.5‐fold) levels and a decrease in the KYNA/QUIN ratio (over −2.5‐fold in neostriatum and over ‐twofold in frontal cortex) were detectable at stage 1 of HD. Qualitatively similar changes were observed in mice transgenic for the full‐length mutant huntingtin.^132^, ^137^ In the advanced stages of HD, a reduction (−1.6‐fold) in KYNA CSF levels was observed.^136^ In the periphery, the baseline L‐kynurenine levels were higher in HD and the difference remained obvious despite tryptophan depletion or loading.^138^ Serum KYNA level in HD was not altered in comparison with control; however, the KYNA/L‐kynurenine ratio was lower.^138^ In a cohort of patients at different stages of HD, the greatest increase in the L‐kynurenine/tryptophan ratio and their overall concentrations was observed among patients possessing more CAG repeats or in those in the later stages of HD.^139^ These observations suggest that changes in the activity of KP, possibly leading to the excessive activity of the neurotoxic arm of the pathway, may have an impact on the development of HD.

In a prospective single‐site controlled cohort study with standardized collection of CSF, blood, and phenotypic and imaging data, performed among 80 participants (20 healthy controls, 20 pre‐manifest HD, and 40 manifest HD), the KP metabolites in CSF and plasma were stable over 6 weeks of observation.^140^ There were no differences regarding basal KYNA, L‐kynurenine, or tryptophan levels. However, an increase in 3‐HK/KYNA ratio was detected in the group of patients with evident HD compared with HD patients at early stage of disease.^140^

Pathologically, high levels of neurotoxic L‐kynurenine metabolites accompanied by a relative lack of neuroprotective KYNA is a consistent finding in HD patients and in animal models of this disease. The deficiency of KYNA and malfunction of the neuroprotective arm of the KP may generate virtually identical consequences as an excessive production of QUIN and other neurotoxic kynurenines. In line with these observations, switching from the neurotoxic branch of the KP, yielding QUIN, to the neuroprotective branch producing KYNA was suggested to bring beneficial effects. Brain changes in KYNA and other KP metabolites can be considered the hallmarks of HD. The encouraging effects of KMO and TDO inhibition in HD models (vide paragraph 4.1.1) are the base for future clinical trials evaluating therapeutic potential of KMO inhibitors.

### 3.2 Parkinson's disease

Parkinson's disease (PD) is a common, progressive neurodegenerative disease, characterized by the gradual loss of dopaminergic brain neurons. Its most characteristic symptoms include resting tremor, limb rigidity, posture and gait instability, and bradykinesia. Loss of dopaminergic neurons in substantia nigra pars compacta and the appearance of intracytoplasmic proteinaceous inclusions, Lewy bodies, are characteristic morphological alterations.^141^ While 10 to 15% of cases represent the familial form of PD, the idiopathic form of disorder prevails.^96^ Disruptions in the ubiquitin‐proteasome and autophagolysosomal pathways, mitochondrial dysfunction, excessive oxidative stress, and enhanced apoptosis were all implicated in the pathogenesis of PD.^142^ Furthermore, disturbed glutamate‐mediated transmission and KYNA deficiency are also among important factors contributing to the development of PD. Neuroprotective and antiparkinsonian effect of glutamate receptor antagonists was demonstrated already 3 decades ago, using various experimental models.^143^, ^144^ In line with these observations, an increase in brain KYNA level, either through the direct application or through increased availability of L‐kynurenine, effectively reduced neurodegeneration and behavioral symptoms in animal models of PD.

Canonical model of PD is based on the administration of lipophilic compound, 1‐methyl‐4‐phenyl‐1,2,3,6‐tetrahydropyridine (MPTP). In 1983, MPTP was discovered as contaminant of street heroin responsible for a rapid development of PD among young addicts.^145^, ^146^ This highly selective neurotoxin causing nigral degeneration, followed by a classical PD‐like behavioral pattern in various species, including rodents and primates, quickly became a valuable research tool.^146^ The mechanisms underlying selective toxicity depend primarily on the glial conversion of MPTP to pyridinium metabolite (MPP+), which, upon release from astrocytes, inhibits neuronal mitochondrial respiratory chain and constitutes a source of free radicals.^147^ Interestingly, MPP^+^ was discovered to inhibit the cortical KAT activity and to reduce KYNA formation *in vitro* in rat cortical slices.^126^ The effect was confirmed i*n vivo*, as MPTP decreased KYNA synthesis and the density of KAT I immunoreactive nigral neurons in mice.^148^, ^149^ Consistently, KYNA pretreatment was shown to reduce the apoptosis of neurons by downregulating Bax expression and maintaining mitochondrial function, in human neuroblastoma cell line exposed to MPP^+^.^150^

Human studies mostly demonstrate that in the brain of PD victims, the metabolism of tryptophan is shifted toward neurotoxic kynurenines with ensuing deficiency of KYNA. Postmortem studies reported diminished KYNA and L‐kynurenine levels in frontal cortex, putamen, and substantia nigra, without change in tryptophan/L‐kynurenine and L‐kynurenine/KYNA ratios, in the brains of PD victims.^151^, ^152^ In caudate and precentral cortical gyrus, KYNA content did not differ from control values.^80^

In the periphery, the results are not consistent. In erythrocytes obtained from PD patients, higher levels of KYNA and enhanced activity of KAT II, but not of KAT I, were detected. In serum, KYNA level remained unchanged, while KAT I and KAT II activities were lower.^153^ Similarly, an increase in L‐kynurenine/tryptophan ratio, depletion of plasma tryptophan level, and increase in L‐kynurenine and KYNA were reported.^154^ Increase in serum KYNA was also observed among patients without dyskinesia, but not in dyskinetic PD patients.^155^

In contrast, the deficiency of KYNA was revealed in a metabolomic study performed on a larger cohort of PD patients. Findings included lower plasma KYNA/L‐kynurenine ratio, higher QUIN level, and increased QUIN/KYNA ratio.^156^ Similarly, lower KYNA, higher QUIN, and an elevated QUIN/picolinic acid ratio in CSF, as well as high 3‐HK in plasma, were detected.^157^

The above data suggest that deficient KYNA synthesis seems to be limited to the brain in the course of PD, whereas in the periphery, the direction of changes in the KP varies and may depend on the stage of disease and the presence of discrete inflammation. Future research should be aimed to analyze in detail the temporal dynamics of peripheral and central KYNA levels in PD. Prominent support to the concept of causal relationship between central KYNA deficiency and PD development comes from the studies utilizing pharmacological compounds to increase brain KYNA and indicating the beneficial effect of such approach in animal models (vide paragraph 4.1.2).

### 3.3 Alzheimer's disease

Alzheimer's disease (AD) is the major cause of age‐related dementia among elderly population. This progressive neurodegenerative disorder leads inevitably to a severe deterioration of cognitive functions and exerts dramatic negative impact on patients’ quality of life. The characteristic neuropathological features of AD include senile plaques composed of beta‐amyloid aggregates and neurofibrillary tangles built from hyperphosphorylated tau proteins.^158^, ^159^ Cholinergic neurons of the forebrain and hippocampal and cortical glutamatergic neurons are among the most affected areas.^160^

In a transgenic mouse model of AD, a decrease in brain KYNA was confirmed.^161^ However, the data on KYNA levels in AD patients are not consistent.^160^, ^162^, ^163^, ^164^ Up to our knowledge, the data from brains of AD patients are very limited. In a small study involving postmortem analyses of specimens obtained from 11 patients with an advanced stage of AD, KYNA concentration was not altered in cortical areas, and increased in putamen (1.92‐fold) and caudate nucleus (1.77‐fold).^160^ In latter structures, elevated KYNA correlated with the KAT I activity.^160^ Lower KYNA levels was also detected in 5 brain structures obtained from AD victims.^83^

Analyses of KYNA content in CSF of AD patients is not conclusive. In mild (N = 41) and moderate‐severe (*N* = 20) AD patients, high KYNA and increased KYNA/tryptophan ratio (both c. 2.7‐fold) were detected.^165^ Similar results were obtained by other groups, reporting 1.7‐fold increase (*N* = 20) vs controls (*N* = 18)^166^ and 1.29‐fold increase (*N* = 40) vs cognitively healthy controls (*N* = 34).^167^ Higher CSF KYNA levels in AD females and significant correlation in the AD group (*N* = 19) of CSF KYNA with sICAM‐1 and CSF P‐tau, but no association with T‐tau or Aβ1‐42, were found.^164^ In contrast, a decrease in CSF KYNA levels among AD patients (−1.3‐fold) and reduction in KYNA content in erythrocytes (−1.54‐fold) and serum (−1.46‐fold) (*N* = 28) were detected by others.^60^, ^168^

In the periphery, patients with AD exhibited a profound (35%) decrease in KYNA in plasma and erythrocytes, although the activity of KAT I and KAT II was not altered.^60^ A similar decrease (−1.48‐fold) of plasma KYNA level in AD patients (*N* = 34) accompanied by an enhanced tryptophan degradation (−1.35‐fold) was reported by others.^169^ The same population of patients manifested higher L‐kynurenine/tryptophan ratio (1.61‐fold), whereas KYNA/L‐kynurenine and 3‐HK/L‐kynurenine ratios were decreased (−1.69‐fold and −1.25‐fold, respectively).^169^ Furthermore, patients had elevated level of serum QUIN indicative of a shift in the peripheral KP toward the neurotoxic metabolites at the expense of KYNA.^169^ In a recent large‐scale metabolic phenotyping study, analyzing urine (*N* = 560) and serum samples (*N* = 354) obtained from clinically diagnosed patients with AD and mild cognitive impairment, lower metabolite concentrations of L‐kynurenine (serum), kynurenic acid (urine), tryptophan (urine, serum), and L‐kynurenine/tryptophan ratio (urine) were reported.^170^

The nature of a decreased KYNA in the periphery among AD patients requires consideration, especially in the view of a well‐established age dependency of serum and brain KYNA levels. AD and dementia affect, in a vast majority, elderly patients. Therefore, AD patients should manifest higher levels of KYNA, which, indeed, are observed in CSF. However, neither in peripheral blood nor in the brain tissue such increases occur. Let us hypothesize that the KP is defective among AD patients, leading to an enhanced formation of QUIN with concomitant decline in KYNA. In such scenario, KYNA levels should be low in periphery, brain, and CSF, which is not a universal finding. However, an important aspect of brain tissue obtained from patients with an advanced AD is evident and prominent widespread neurodegeneration. Neuronal loss results in inflammation and subsequent astrogliosis. Increased number of astrocytes is directly associated with an enhanced activity of KATs.^171^, ^172^, ^173^ Thus, hyperactivity of KATs and subsequent overproduction of KYNA developing after the occurrence of neuronal loss would not be perceived as a cause, but rather as one of the consequences of neurodegeneration and reactive gliosis.

A support to the concept of deficient KYNA formation as one of the causative factors in AD comes from the elegant study in a transgenic mouse model of AD. The pharmacological manipulation aimed to increase KYNA level prevented a number of behavioral and neuropathological changes in this model^161^ (vide paragraph 4.1.3).

The potential role of disturbed KYNA formation in the pathogenesis of dementias is not fully understood. It is important to note that disproportionately high KYNA production, in our opinion secondary to the neuronal loss, may be aimed to further prevent the death of neurons in AD. Unfortunately, at high concentrations KYNA may act as a double‐sword and actually impair working memory and contextual learning.^174^, ^175^, ^176^, ^177^ An increase in error frequency has been reported in rats treated intraperitoneally with L‐kynurenine and manifesting high levels of brain KYNA, produced *de novo* within the brain from its precursor.^116^ Similarly, adult rats treated throughout their adolescence with L‐kynurenine exhibited deficits in contextual fear memory, a novel object recognition memory, but not cue‐specific fear memory.^175^ Adult rats exposed pre‐ and postnatally (gestation day 15‐postnatal day 21) to L‐kynurenine manifested a threefold increase in forebrain KYNA levels, a 2.5‐fold increase in prefrontal cortex KYNA, and deficits in initial reversal learning and extra‐dimensional shift.^176^

Hence, KYNA, a metabolite of KP with neuroprotective effects at physiological concentrations, may exacerbate cognitive dysfunction and memory impairment in AD. However, as discussed above, an increase of brain KYNA levels most probably results from and is not a cause of neurodegeneration. In order to clarify this issue, longitudinal studies assessing the level of KYNA prior to and during the occurrence of overt symptoms of AD and dementia should be performed.

## 4 THERAPEUTIC PERSPECTIVES OF INCREASING KYNA LEVELS IN NEURODEGENERATIVE DISORDERS

In the past, given the very limited penetration of KYNA through the BBB and its rapid removal from the brain and body, the use of KYNA in the treatment of neurodegenerative diseases seemed virtually impossible.^178^ Various attempts aimed to refine the bioavailability of KYNA have brought promising results. The most successful approaches are based on the use of KYNA analogs penetrating through the blood‐brain barrier, or modulation of the KP aimed to increase the concentration of KYNA substrate, L‐kynurenine, in the periphery through an inhibition of selected key enzymes. The latter approach results in an enhanced availability of L‐kynurenine for brain KYNA synthesis, as this KP metabolite easily enters central compartment. As the current reports on the KYNA therapeutic abilities *in vivo* seem rather optimistic, bypassing the main obstacle by improving its bioavailability may be a milestone in introducing KYNA to the treatment of neurodegenerative diseases.

### 4.1 Inhibition of KMO

Modulation of the KP‐controlling enzymes is a crucial step toward the increased production of neuroprotective metabolites with a simultaneous reduction in neurotoxic QUIN and 3‐HK in the brain. Due to the fact that astrocytes do not express KMO, the major astrocytic product of tryptophan catabolism is KYNA.^179^ In contrast, microglia and macrophages convert tryptophan along both arms of the KP—neuroprotective and neurodegenerative.^70^ In such scenario, proinflammatory environment, consistently implied as one of the factors contributing to the development of neurodegeneration, leads to an ample production of neurotoxic kynurenines, such as QUIN or 3‐HK.^180^ Thus, inhibition of KMO allows astrocytes to retain more of L‐kynurenine and to produce larger amounts of KYNA, sufficient to antagonize the glutamate and QUIN excitotoxicity. Indeed, it is broadly documented that KMO activity is crucial for directing the metabolic fate of L‐kynurenine, and thus influences the QUIN/KYNA ratio the most.^181^ A number of KMO inhibitors were synthesized and tested in various experimental models.^182^, ^183^ The initial studies were carried out prior to the identification of crystal structure of KMO; thus, the design of earliest inhibitors was based on the structure of L‐kynurenine.^182^ One of the first KMO inhibitors used experimentally was nicotinylalanine.^184^, ^185^ The development of selective KMO inhibitors started in the 1990s, with introduction of m‐nitrobenzoylalanine (mNBA) showing the IC_50_ = 0.9 μM.^186^ Experimental administration of 400 mg/kg of mNBA to rats resulted in a substantial increase of L‐kynurenine and KYNA levels—13‐ and fivefold in the brain, fivefold and 2.4‐fold in the blood, and sixfold and 3.5‐fold in the liver, respectively.^183^ mNBA served as a lead compound for the synthesis of novel inhibitors belonging to 4‐phenyl‐4‐oxobutanoic acids, such as (R, S)‐3,4‐dichlorobenzoylalanine (FCE 28833).^187^ After intraperitoneal treatment with FCE 28833A in rats (400 mg/kg), extracellular brain KYNA levels remained significantly elevated (over 30‐fold) for at least 22 h.^187^ Modifications of FCE 2883 have brought development of PNU‐168754 and UPF‐648, a potent and selective KMO inhibitors (IC_50_ = 40 and 20 nM, respectively).^188^ In gerbils, administration of UPF‐648 evoked remarkable increase of L‐kynurenine and KYNA levels in brain and plasma.^189^ Another class of potent inhibitors comprises sulfonamides, including Ro 61–8048 (3,4‐dimethoxy‐N‐[4‐(3‐nitrophenyl) thiazol‐2‐yl] benzenesulfonamide; IC_50_ = 37 nM).^188^ Ro 61–8048 provided neuroprotection in the rat and gerbil ischemia models, displayed antiepileptic activity against electroshock‐induced seizures in mice and rats, and reduced the cerebral QUIN accumulation in mice subjected to immune activation.^66^, ^190^, ^191^, ^192^ Due to the relative instability of Ro 61–8048, its slow‐release prodrug form, 2‐(3,4‐dimethoxybenzenesulfonylamino)‐4‐(3‐nitrophenyl)‐5‐(piperidin‐1‐yl) methylthiazole (JM6), has been developed.^161^ Next class of KMO inhibitors was developed after establishing the crystal structure of the *Saccharomyces cerevisiae* enzyme (ScKMO) and human enzyme (hKMO).^193^, ^194^ These compounds include aryl pyrimidine carboxylic acids, 3,4‐dichlorohippuric acid, or 5‐(3‐nitrobenzyl)‐1H‐tetrazole.^182^

#### 4.1.1 Huntington's disease

Reestablishment of the physiological ratios between KP metabolites and shifting of the KP toward neuroprotective compounds may yield a potentially therapeutic effect in HD. Indeed, protective action of KMO inhibition was demonstrated in various experimental models of HD. Both genetic (*cinnabar* and *vermillion* mutations) and chemical (UPF‐648) inhibition of KMO attenuated neuronal loss in *Drosophila melanogaster* HD models.^195^ UPF‐648 ameliorated the QUIN‐induced excitotoxic neuronal damage in transgenic *mKAT II* ^−/−^ mice and prevented degeneration of the rhabdomeres (photoreceptor neurons) in fruit fly HD model.^195^ In the transgenic model of HD, oral administration of KMO inhibitor was linked with the animal life span prolongation, neuroprotection, and reduced glial activation.^161^ Inhibition of KMO activity with CHDI‐340246 diminished the brain formation of 3‐HK and QUIN, elevated L‐kynurenine and KYNA levels, and restored the electrophysiological alterations. However, chronic application of CHDI‐340246 did not modify behavioral phenotypes or natural progression in mouse models of HD.^196^ Similar, favorable effects were obtained with an inhibitor of TDO in a fruit fly model of HD. Improved locomotor performance, extended life span, and reduced neurodegeneration in Alzheimer's model flies were linked with an increased KYNA/3‐HK ratio.^197^

#### 4.1.2 Parkinson's disease

Neuroprotective and antiparkinsonian effects of glutamate receptor antagonists are well documented.^143^, ^198^ In line with these data, either administration of KYNA itself or use of pharmacological tools increasing the availability of L‐kynurenine and its conversion to KYNA may reduce neuronal loss and behavioral symptoms in experimental PD models.^185^, ^199^, ^200^, ^201^, ^202^ Modifications of tryptophan metabolism seem to exert dual therapeutic benefit in PD, neuroprotection, and prevention of L‐DOPA–induced motor side effects.^203^

Direct application of KYNA into the medial segment of the globus pallidus reduced the behavioral symptoms in MPTP‐induced PD model.^199^ Similarly, in monkeys with hemiparkinsonism induced by unilateral, intraarterial administration of MPTP, KYNA infusion into the contralateral globus pallidus internus alleviated the disease symptoms.^200^ The intracerebroventricular infusion of nicotinylalanine, inhibiting kynureninase and L‐kynurenine hydroxylase activity, combined with L‐kynurenine and probenecid, an inhibitor of organic acid transport, substantially increases KYNA level in rodent brain. This approach was used to raise KYNA content in rat substantia nigra and appears to be sufficient to protect neurons from QUIN‐induced toxicity.^185^ Furthermore, intraperitoneal administration of L‐kynurenine and probenecid was demonstrated to reduce the 6‐hydroxydopamine–evoked neuronal damage and behavioral alterations.^204^ In a primate model of MPTP‐induced parkinsonism, orally administered KMO inhibitor, Ro 61–8048, increased central L‐kynurenine and KYNA levels. This treatment improved the L‐DOPA‐induced dyskinesias without alteration of drug's antiparkinsonian efficacy.^205^ Ro 61–8048 also reduced L‐DOPA motor side effects without affecting PD exacerbation.^159^ In QUIN‐lesioned striata, KMO inhibitor UPF‐648 decreased conversion of L‐kynurenine to downstream neurotoxic metabolites, 3‐HK and QUIN (by 77% and 66%, respectively) and moderately raised KYNA synthesis (by 27%).^95^ On the contrary, intrastriatal application of UPF‐648 in naïve rats reduced 3‐HK synthesis (by 64%) without change in KYNA formation.^98^

In a fly model of PD, TDO inhibition evoked dramatic reduction in the 3‐HK/KYNA ratio, mainly due to increased synthesis of KYNA, with ensuing amelioration of disease phenotypes.^197^

Prolonged systemic administration of the KMO inhibitor Ro 61–8048 reduced L‐DOPA–induced dyskinesias in 1‐methyl‐4‐phenyl‐1,2,3,6‐tetrahydropyridine (MPTP)–treated monkeys with overt symptoms of parkinsonism.^206^ Immunosuppressive drug, FK506, shown to exert neuroprotective effects in experimental PD models is also able to increase KYNA formation and to prevent the MPP^+^‐induced decline in KYNA synthesis.^207^, ^208^

Experimental data consistently imply that shift of the KP to neurotoxic branch producing 3‐HK and QUIN formation, with relative or absolute deficiency of KYNA, is an important factor contributing to the development and progress of PD. We are still awaiting the synthesis of more precise pharmacological tools, able to modulate the KP within basal ganglia in a selective manner, which may become a promising therapeutic option for PD.

#### 4.1.3 Alzheimer's disease

Only limited studies exploited KMO inhibitors as possible therapy for AD. Chronic oral therapy with JM6, inhibitor of KMO, was demonstrated to rise brain KYNA, due to de novo synthesis of the compound from L‐kynurenine, and to reduce extracellular glutamate in a transgenic mouse model of AD.^161^ JM6 did not exert significant effects on Aβ plaque formation; however, it prevented spatial memory deficits. The compound also extended life span, prevented synaptic loss, and decreased microglial activation.^161^

#### 4.1.4 KMO inhibitors—limitations

Despite a large therapeutic potential, there are important drawbacks of some currently available KMO inhibitors. Certain compounds, such as mNBA and UPF‐648, were found to act as uncouplers of NADPH oxidation, which may actually potentiate neuronal loss via generation of cytotoxic hydrogen peroxide.^209^ Therefore, precise design of novel compounds, effectively increasing brain KYNA levels, yet devoid of harmful production of free radicals, remains an important goal in the development of drugs against neurodegenerative disorders.

### 4.2 Analogs and prodrugs of KYNA

The goal of creating new KYNA analogs and prodrugs was to overcome the obstacle of poor BBB penetration by KYNA itself and to synthesize precursors that preferentially would not be metabolized to neurotoxic kynurenines. As a result, chlorokynurenines, including 4‐chlorokynurenine and 4,6‐dichlorokynurenine, emerged, meeting the above criteria, including fast delivery into the CNS and, once in the brain parenchyma, an easy conversion to potent NMDA antagonists acting at the glycine site, 7‐chlorokynurenic acid, or 5,7‐dichlorokynurenic acid.^210^ Similar parameters characterize also esterified analogs and esterified 4‐amino analogs.^94^ Another approach to improve the BBB penetration was based on utilizing D‐glucose ester of 7‐chlorokynurenic acid. The conjugate manifests improved BBB penetration as a result of an active transport by the glucose transporter GLUT1.^211^ Indeed, systemic administration of the conjugates resulted in an anticonvulsant effect in mice affected by NMDA‐associated seizures.

#### 4.2.1 Huntington's disease

Intraperitoneal injection of KYNA derivative, N‐(2‐N,N‐dimethylaminoethyl)‐4‐oxo‐1H‐quinoline‐2‐carboxamide hydrochloride (SzR72), diminished hypolocomotion, increased survival time, and provided striatal neuroprotection in transgenic N171‐82Q mice, without any major adverse effects.^212^ Under *in vivo* conditions, both KYNA and SzR72 not only did not reduce but also actually enhanced the induction of long‐term potentiation (LTP).^213^ The absence of memory impairment may result from the selective block of extrasynaptic NMDA and α7nACh receptors, while sparing the synaptic NMDA‐mediated currents.^213^

#### 4.2.2 Alzheimer's disease

Various KYNA analogs were synthesized and subsequently tested for their biological utility in the transgenic *Caenorhabditis elegans* line GMC_101_ with fully expressed Aβ_42_, AD model.^214^ Three promising analogs representing a complex anti‐AD mechanism (free radical scavenger, AChE inhibitor, binding the mGluR5 and NMDA receptors and inhibiting the progression of Aβ fibrillation) include methyl 4‐hydroxy‐8‐methoxy‐5‐nitroquinoline‐2‐carboxylate, methyl 8‐amino‐4‐hydroxy‐6‐methoxyquinoline‐2‐carboxylate, and methyl 5‐amino‐4‐hydroxy‐8‐methoxyquinoline‐2‐carboxylate. The last two compounds exhibited fine permeability through the BBB model (cell‐based MDR1‐MDCKII), thus allowing to bypass the greatest limitation in KYNA bioavailability. The latter of the three analogs also showed a neuroprotective effect against Aβ‐related toxicity.

#### 4.2.3 Parkinson's disease

Up to our knowledge, KYNA analogs were not studied in experimental models of Parkinson's disease. However, a number of compounds able to increase KYNA levels, through mechanisms distinct from interference with KP, successfully ameliorated L‐DOPA–induced dyskinesias. One of the interesting therapeutic options for PD seems to be the antiepileptic drug zonisamide, shown to reduce motor symptoms in patients with L‐DOPA–induced dyskinesias.^215^ Zonisamide, apart from his broad pharmacological effects including inhibition of voltage‐gated sodium channels, T‐type calcium channels, and monoamine‐oxidase, has been shown to increase KYNA production.^216^ Short and long exposure of the astrocytes to zonisamide increased the production of KYNA and other metabolites: xanthurenic acid and cinnabarinic acid, both with the properties of endogenous metabotropic glutamate receptor agonists (II and III groups, respectively).^216^

FK506, a neuroimmunophilin ligand with immunosuppressive properties, used in the PD therapy, has been demonstrated both to enhance the cortical KYNA production and to restore the production of KYNA inhibited by the MPP+or 3‐NP.^207^ Long‐term administration dose‐dependently increased dopaminergic neuron survival in an α‐synuclein–based rat model of Parkinson's disease.^217^

## 5 CONCLUDING REMARKS

Alterations in central and peripheral KYNA synthesis have been demonstrated in the course of neurodegenerative diseases such as Alzheimer's, Parkinson's, or Huntington's disease, and deficiency of KYNA appears to contribute, at least in part, to the pathogenesis of neuronal loss. So far, accumulated data failed to show repeatedly the reliable correlation between peripheral and brain KYNA levels, as recently reviewed in a systematic review.^218^ However, despite conflicting results, blood KYNA levels were linked with clinical symptoms and treatment response in psychiatric patients, as well as with observed neuroanatomical abnormalities and glial activity.^218^ It would be optimal to combine the experiments involving simultaneous measurements of KYNA in blood and CSF, in future research involving experimental animal models, and in human studies aimed to elucidate KYNA contribution to neurodegeneration.

The current state of research is to develop promising experimental methods of manipulating the KP in humans without side effects, aimed to inhibit the KP part responsible for the synthesis of QUIN with concomitant stimulation of KYNA synthesis. Potential treatments include inhibitors of certain KP enzymes, as well as new prodrugs and analogs of KYNA penetrating via the blood‐brain barrier and thus able to enhance the KYNA‐induced block of the glycine site within the NMDA receptor complex. Emerging therapies may become an important route in the treatment of neurodegeneration, especially when the targeting of specific brain regions will be possible. Selectivity seems vital, especially considering that KYNA, apart from being neuroprotective, when produced in excessive quantities may also hamper cognition. The use of KYNA, alone or in combination with pharmacological tools precisely influencing specific populations of neurons, is awaiting to become a significant therapy for neurodegenerative disorders.

## CONFLICT OF INTEREST

The authors declare that they do not have any conflicts of interest.
